# Growth Abnormalities in Children with Type 1 Diabetes, Juvenile Chronic Arthritis, and Asthma

**DOI:** 10.1155/2014/265954

**Published:** 2014-02-04

**Authors:** Cosimo Giannini, Angelika Mohn, Francesco Chiarelli

**Affiliations:** ^1^Department of Pediatrics, University of Chieti, Ospedale Policlinico, Via dei Vestini 5, 66100-Chieti, Italy; ^2^Center of Excellence on Aging, “G. D'Annunzio” University Foundation, University of Chieti, Via dei Vestini 5, 66100-Chieti, Italy

## Abstract

Children and adolescents with chronic diseases are commonly affected by a variable degree of growth failure, leading to an impaired final height. Of note, the peculiar onset during childhood and adolescence of some chronic diseases, such as type 1 diabetes, juvenile idiopathic arthritis, and asthma, underlines the relevant role of healthcare planners and providers in detecting and preventing growth abnormalities in these high risk populations. In this review article, the most relevant common and disease-specific mechanisms by which these major chronic diseases affect growth in youth are analyzed. In addition, the available and potential targeting strategies to restore the physiological, hormonal, and inflammatory pattern are described.

## 1. Introduction

During childhood and adolescence, the longitudinal growth of bones represents one of the most relevant changes of the body composition [1]. Bone growth occurs at different rates and results from complex mechanisms involving a multitude of regulatory hormones. These events are directly influenced by the interaction between genetic and environmental factors [1–4]. Nutritional status represents one of the most relevant factors affecting these interactions. However, several other factors, and especially chronic diseases, might also strongly modulates these complex mechanisms. In fact, chronic diseases, by directly or indirectly modulating bone and hormonal status, may affect growth and final height of subjects with a disease onset during childhood or adolescence. Several lines of evidence have clearly shown that growth is often impaired in children and adolescents with type 1 diabetes (T1D), juvenile idiopathic arthritis (JIA), and asthma represents the one of most common chronic inflammatory disease in childhood.

A complete knowledge of the physiological events leading to a regular growth during childhood and adolescence and especially of those alterations developed in these populations at high risk of growth impairment, is needed in order to allow a physiological growth during this critical phase of development and the attainment of an appropriate final height.

## 2. Growth and Development

Longitudinal bone growth represents a complex process involving a multitude of regulatory mechanisms strongly influenced by growth hormone (GH) [1–4]. GH has a pulsatile secretion with age-dependent concentrations. In fact, GH concentrations tend to be low during the prepubertal period and characteristically increase at puberty and then decrease again during adulthood. Most of the growth promoting effects related to GH are mediated through the actions of peptides, the insulin-like growth factors-I (IGF-I), and IGF-II which are mainly secreted by the liver. IGFs circulate bound to different insulin-like growth factor binding proteins (IGFBPs). Among them, IGFBP-3 represents the major circulating form and its concentrations have been shown to be GH dependent. These binding proteins play relevant functions in the regulation of the GH/IGFs axis by prolonging the half-life of IGFs and by carrying IGFs to the target tissues making a ternary complex with the acid labile subunit (ALS) [5–7]. Although IGFs have several metabolic effects, the most relevant role of these proteins is to promote length increase in the long bones by regulating growth plate chondrocyte proliferation, maturation, and hypertrophy, as well as to induce matrix synthesis and degradation. Insulin represents one of the most important regulators of this system. Several studies have shown that adequate insulin secretion and normal portal insulin concentrations are needed to support normal serum concentrations of IGFs and IGFBPs and indirectly to promote growth. Of note, there is strong evidence suggesting that several inflammatory cytokines, and especially interleukin-1*β* (IL-1 *β*), tumor necrosis factor-*α* (TNF-*α*), and interleukin-6 (IL-6) [8], may act individually or in combination to affect child growth. These molecules may act through systemic mechanisms and/or local action at the level of the growth plate of long bones [8]. Finally, treatment adopted in several chronic diseases, mainly chronic corticosteroid treatment, may strongly affect bone metabolism and consequently exert negative effects on growth of children and adolescents.

Although peculiar mechanisms might be involved in each chronic disease, important contributors to the occurrence of impaired growth are the disease related treatment or the disease itself or a combination of both. These factors, along with other relevant determinants, including disease duration, severity and activity of the disease, or poor nutrition and reduced physical activity, represent the main determinants of growth impairment in chronic diseases in children and adolescents [9].

## 3. The Burden of Chronic Diseases in Children and Adolescents

The incidence of type 1 diabetes (T1D) is increasing worldwide at an annual rate of around 3–5%, particularly in children under the age of 5 years [10, 11]. Approximately 50–60% of patients with T1D are diagnosed before the age of 15 years, and in most Western countries T1D accounts for over 90% of cases of childhood and adolescent diabetes [12]. Based on data from the International Diabetes Federation, in 2011, there were around 490 thousands of children (age of 0–14 years), with 77.8 thousands of newly diagnosed cases [12]. There are wide variations in the incidence rates of T1D across countries, with the lowest incidence reported in China and Venezuela (0.1 per 100,000 per year) and the highest incidence in Finland and Sardinia (37 per 100,000 per year) [10]. Recent epidemiological data from Europe indicate a number of 15,000 new cases of T1D in people younger than 15 years, and this number is predicted to rise to 24,400 in 2020 [11].

JIA represents a chronic inflammatory disease affecting the joints and represents the most common chronic rheumatic disease during childhood [13]. Recent reports indicate an annual incidence ranging from 5 to 18 per 100,000 children with an overall prevalence of 30–150 per 100,000 [13].

Asthma represents the most frequent chronic inflammatory disease in childhood [14, 15]. The World Health Organization (WHO) includes asthma among the major chronic disorders, representing a worldwide public health priority [14, 15]. Over the past decades, the prevalence of Asthma has steadily increased reaching epidemic proportions. The CDC National Surveillance for asthma revealed that its prevalence in children has risen from 3.5% to 7.5% over a period ranging from 2001 to 2003. As many as 10–15% of boys and 7–10% of girls may have asthma during childhood [16].

The high incidence and in particular the peculiar onset of these chronic diseases mainly during childhood and adolescence represent a health priority for healthcare planners and providers. A complete knowledge of the physiological events leading to growth alterations in children and adolescents with these chronic diseases is crucial in order to allow a growth as physiological as possible and attainment of expected final height.

## 4. Growth in Children and Adolescents with Type 1 Diabetes

A large amount of data have clearly documented a central role of insulin as one of the main regulators of GH/IGFs axis. Insulin regulates the expression of GH receptors in the liver and affects IGFs and IGFBPs synthesis by modulating the GH postreceptor events [17–19]. As well, by negatively modulating gene expression and secretion of IGFBP-1, insulin significantly increases IGF-I bioactivity, which is negatively regulated by IGFBP-1 concentrations [20, 21]. By impairing this complex regulatory physiology, low portal insulin concentrations, documented in children with T1D, result in GH hypersecretion, low circulating levels of IGF-I and IGFBP-3, and high circulating levels of IGFBP-1. Studies in newly diagnosed subjects with T1D have demonstrated decreased circulating concentrations of GH binding protein, which are considered a putative index of GH receptor number [22–24]. As well, insulin therapy has been shown to restore GH binding protein concentrations, although levels remain lower than those found in normal subjects [24]. In turn, all these alterations related to portal insulin deficiency result in an increased risk of developing growth failure [25], as documented in youth with Mauriac syndrome [26]. This rare and severe form of growth failure documented in T1D is characterized by hepatomegaly, growth and puberty delay, and the presence of elevated transaminases and serum lipids levels. The development and progression of bone alterations are variable between subjects being strongly affected by several factors (Table 1), including age at onset and duration of the disease, sex, and mainly glycemic control. Recent advances in insulin regimes and diabetes-related technologies have significantly reduced the occurrence of extreme forms of growth alteration in children and adolescents with T1D. In fact, insulin regimes, new insulin analogs, and new technologies available for the treatment of subjects with T1D have led to more physiological circulating insulin concentrations, thus improving GH/IGFs alterations. However, although relevant progresses have been reached in the field of treatment of children and adolescents with T1D, Mauriac syndrome and alterations of GH/IGFs axis can still be documented, thus requiring a complete characterization of the underling mechanisms related to impaired growth in this high risk population.

Several studies have clearly documented impaired prepubertal and pubertal growth in children and adolescents with T1D [27–30]. Unlike height at diagnosis has been reported to be normal in some studies but impaired in other [31–34], or even increased in some studies [35–39]; consistent results have reported a decline in height SDS from diagnosis to the onset of puberty in children with T1D. Data reported by Brown et al. demonstrated a change in height SDS between diagnosis and the onset of the pubertal spurt of 2.02 (range from 0.48 to 2.10) and a 0.06 SDS mean loss of height per year between diagnosis and the onset of puberty [35]. Although the timing and duration of the pubertal growth spurt are normal [35], a blunted pubertal growth spurt has been documented, which, in adolescents with T1D, seems to be associated with a reduced peak height velocity SDS [32, 35, 40, 41]. Of note, these effects appear to be also influenced by sex and age at diagnosis. In fact, several studies have reported a lower mean peak height velocity in girls than in boys [35, 42] and an association between poor growth and younger age at onset [35, 43].

The severity of the impaired prepubertal and pubertal growth is mainly related to glycemic control and to the adopted insulin regimes. Children with T1D and poor metabolic control have a significantly lower growth velocity [44] and lower IGF-I levels than those with adequate metabolic control [27–30, 45]. Similarly, these subjects show low median IGFBP-3 serum concentrations [46–48], with a negative correlation between growth indexes and both HbA1c levels [28, 29], and serum insulin concentrations achieved by exogenous insulin therapy [49, 50]. As shown in a recent study evaluating a large population of 22,651 children with T1D from specialized centers in Germany and Austria, the entire cohort “lost” 0.41 SDS during the course of the disease [51]. Of note, a negative and significant association between impaired growth and the degree of metabolic control was clearly shown. In fact, in those subjects with mean HbA1c levels >8.0%, the mean near adult height was −0.31 SDS which was significantly higher than that reported in those subjects with a HbA1c level <7.0% (near adult height 0.03 SDS) [51]. Danne et al. showed a direct correlation between increased HbA1c levels and standing height SDS reduction [43]. Of note, the effects of poor metabolic control on growth in subjects with T1D are further enhanced during puberty. In fact, similarly to what documented in healthy adolescents, also in subjects with T1D puberty is associated with a reduction in insulin sensitivity, which might negatively influence growth and height gain [50, 52].

The occurrence of impaired growth after diagnosis and during puberty is associated with abnormalities in the hypothalamus-pituitary-IGF-I axis [27, 28]. IGF-I levels were reported to be reduced in both girls and boys with T1D compared to control subjects [29, 30, 40, 53, 54] and are associated with low serum concentration of IGFBP, and specifically levels of the large molecular weight IGFBP-3 [47, 48]. As well, glycemic control strongly influences IGF-I levels after the onset of puberty [30]. In both sexes, circulating IGF-I levels are closely related to insulin dose [54–56] and tend to be restored by adequate insulin therapy [50, 57].

Improvements in diabetes care and management, with the implementation of newer insulin regimens and analogs, have substantially improved growth in children with T1D by restoring GH-IGF-I axis abnormalities. In a study by Chiarelli et al., the relevant role of insulin regimens and in particular of intensive therapy (using four daily insulin injections) from the onset of diabetes in preventing growth alteration in children and adolescents with T1D has been well described [58]. In this study, a group of thirty male children and adolescents with T1D with different pubertal status (prepubertal, pubertal, and postpubertal) were enrolled and compared with a group of 30 age, sex, and pubertal age matched healthy subjects with the aim of detecting differences in height and in serum concentrations of IGF-1 and IGFBP-3 [58]. Results showed that compared with control subjects IGF-I and IGFBP-3 serum concentrations were within the normal range in the three diabetic groups, suggesting normal function of the GH-IGFI axis in children with T1D. Of note, the resulting physiological GH-IGF-I axis induced a completely normal growth in all the three groups. In fact, no significant difference in terms of height SDS was documented in the three groups compared with control subjects [58]. Thus, these results suggest that intensive insulin therapy starting from the onset of diabetes might prevent the development of abnormalities of the GH-IGF-I-IGFBP-3 axis, likely allowing normal IGF-I and IGFBP-3 levels and physiological growth in children and adolescents with T1D [58]. Although Mauriac syndrome represents an uncommon ancient complication described in children and adolescents with T1D, it still exists, as documented in relatively recent reports particularly in adolescent females [59]. Thus, healthcare planners and providers may be aware of this extreme form of growth failure and especially of the molecular mechanisms characterizing growth alteration in children and adolescents with T1D. This is of utmost importance since most of the clinical features are reversible with better glycemic control and appropriate insulin management.

## 5. Growth in Children and Adolescents with Juvenile Idiopathic Arthritis

**Figure 1:**
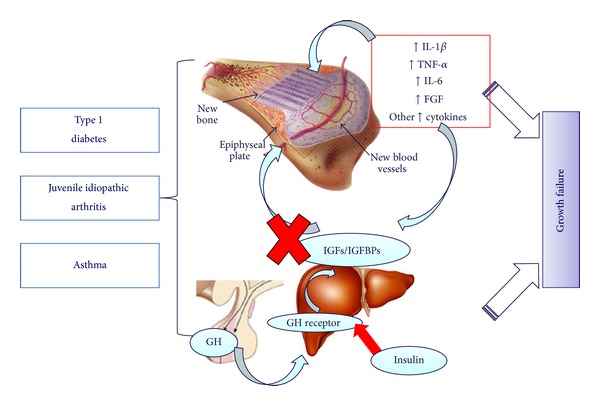
Common factors contributing to growth failure in children and adolescents with type 1 diabetes, juvenile idiopathic arthritis and asthma. GH: growth factor; IGFs: insulin-like growth factors; IGFBPs: insulin-like growth factor binding proteins; IL-1 *β*: interleukin-1*β*; TNF-*α*: tumor necrosis factor-*α*; IL-6: interleukin-6; FGF: fibroblast growth factor.

Studies in children and adolescents with chronic inflammatory diseases and especially JIA have largely documented an increased risk of developing growth alterations during childhood and adolescence [9]. Growth alterations are often characterized by general growth retardation which may range from mild decreases in growth velocity to severe forms of short stature [8]. Although impaired growth is often documented, affected limb may also present local acceleration of growth [8]. The development and progression of bone alterations is variable between subjects being strongly affected by several factors (Table 1) such as the degree, extent and duration of disease activity, age at onset and mainly corticosteroid treatment, undernutrition, reduced physical activity, and proinflammatory cytokine levels (IL-1*β*, TNF-*α*, and IL-6) [60, 61]. Growth might also be influenced by some additional factors (such as vitamin D metabolites, sex steroids, parathyroid hormone-related peptide, fibroblast growth factor, bone morphogenic proteins, and circulating and local expression of the transforming growth factor superfamily proteins) which can act systemically and/or locally with an autocrine/paracrine effect [9, 62] (Table 1 and Figure 1). Although different, these factors may all alter bone homeostasis either by directly affecting growing bone plate or by modulating GH/IGFs axis, at one or more levels, centrally or peripherally [8].

The form of JIA represents an important factor affecting the rate of occurrence of growth failure, being short stature documented in roughly 10.4% and 41.0% of children and adolescents with the polyarticular and systemic forms of JIA, respectively [63–65]. The association between different forms of arthritis and the degree of growth impairment is clearly reported in a longitudinal study evaluating, for a period ranging from 5 to 18 years [61], a group of 67 children affected by different form of JIA. In this study, a progressive decline in height velocity over time was documented in the group with the systemic form. Similarly, in those children with the polyarticular form, a modest decline in height velocity was documented only during the first 5 years of followup, although subjects showed a subsequent tendency towards normalization [61]. In contrast, a relatively stable height velocity during the follow-up period was documented in children with the pauciarticular form of JIA [61].

Long-term steroid treatment has been shown to acutely and chronically affect growth in children and adolescents with JIA. In fact, in a high percent of subjects (~87%) [64], systemic steroids therapy for longer than 12 months has been shown to be significantly associated with deviations of adult height from midparental height [66]. In these subjects, systemic steroid treatment is also associated with a blunted catch-up growth (~30%), documented even after the remission of the disease and particularly even after glucocorticoid therapy has been interrupted. However, several data suggest that impaired growth is a reversible alteration, as documented by progressive normalization of growth velocity to predisease values if the disease activity is well controlled [67] or after the disease remission [68].

During the last decade, the incessant growing of knowledge on the effects of several cytokines associated with JIA has opened new perspective in the field of chronic inflammatory diseases with the aim of restoring growth abnormalities. There are emerging data suggesting that by acting through systemic mechanisms (IL-6) or local action at the level of the growth plate of long bones (TNF-*α* and IL-1*β*), cytokines strongly modulate growth in children and adolescents with JIA [8, 69, 70].

Animal and *in vivo* models of chronic inflammatory disease have shown a significant association between IL-6 concentration and growth retardation [61, 63, 71], mainly characterized by an impaired growth velocity [72]. As shown in transgenic mouse lines and also supported in human studies [73, 74], these effects seem to be related to a peculiar ability of IL-6 to induce an increased proteolysis of serum IGFBP-3, which in turn results in decreasing IGF-1 half-life and its accelerated clearance.

Relatively recent studies have also postulated a direct effect of IL-6 on growth plate chondrocytes. By using murine cell lines (ATDC5), Nakajima et al. [75] were able to show that IL-6 negatively modulates the expression of type II collagen, aggrecan, and type X collagen and inhibits cartilaginous nodule formation, a marker of neochondrogenesis in mesenchymal-cell cultures [75]. Consistent results on a potential role of IL-6 in bone homeostasis have been also reported by De Benedetti et al. [76] using the skeletons of growing prepubertal mice exposed to high circulating IL-6 levels since birth. Promising results on the possibility to reverse bone alteration documented in children with JIA are reported in a study from Nakajima et al. [77]. By evaluating skeletal abnormalities in 201 healthy children with JIA aged 0–16 years, authors showed decreased serum cartilage oligomeric matrix protein (COMP) and bone alkaline phosphatase (BAP) concentrations, which, respectively, reflect chondrocytes turnover and osteoblastic activity, and increased serum metalloproteinase-3 (MMP-3), which represents a predictor of joint destruction and disease activity. More importantly, tocilizumab treatment was able to counteract these effects suggesting a potential reversibility of this alteration as a result of the successful inhibition of inflammation under treatment with anti-IL-6 [77].

In contrast to IL-6 effects which are mainly related to GH/IGFs axis, IL-1*β* and TNF-*α* actions are mainly related to the bone resulting in a significant inhibition of the expression of a number of genes encoding chondrocyte-specific matrix molecules, including aggrecan, collagen types IX and XI [62, 69, 78]. Of note, several studies have shown an additive and synergistic effect of the two cytokines [69, 70, 79], which seems to be partially explained by a shared post-receptorial pathway mainly related to the MAPK-signaling pathways [80]. Furthermore, in growth plate chondrocytes, these cytokines have been shown to modulate IGF-1 signaling pathway [62] through different mechanisms: by down-regulating IGF-1 receptor expression [62]; by modulating IGF-1 receptor affinity [81] in articular cartilage; by inhibiting the intrinsic tyrosine kinase activity of the IGF-1R [82, 83]; by inducing IGF-1 resistance through the modulation of IRS-1 phosphorylation [62].

Therefore, a major role played by the main proinflammatory cytokines is now established which may act individually or in combination to affect child growth [8]. Improvement of new therapeutic strategies in children and adolescents with JIA, and especially of the new cytokine inhibitors, may also play a key role in restoring the growth abnormalities often associated in children and adolescents with chronic inflammatory diseases. In fact, new available treatment for JIA has been shown to restore circulating levels of several key factors involved in growth abnormalities documented in this high risk population. In this perspective, encouraging data have been reported by De Sanctis et al. in a recent study evaluating the effect of 1-year treatment with the antitumor necrosis factor-*α* (TNF-*α*) drug etanercept on lipid profile and oxidative stress in children and adolescents with juvenile idiopathic arthritis [84]. In this study by evaluating a group of thirty children with JIA (22 females; mean age 12.3 ± SD 5.7 years), authors clearly showed that anti-TNF-*α* therapy for JIA is associated not only with a beneficial effect on clinical disease activity and inflammatory indexes, but also with improved lipid profile and oxidative stress. These findings suggest that TNF-*α* blockers might reduce atherosclerotic risk in children with JIA [84].

Further studies are required to completely characterize the long-term effects of the new cytokine inhibitors and especially their ability to prevent the impaired systemic and local growth alterations associated with these important proinflammatory cytokines.

## 6. Growth in Children and Adolescents with Asthma

Since 1940, several authors have reported an association between asthma and inhibition of linear growth. It has been observed that, despite treatment, moderate and severe asthma are responsible for a delay in pubertal growth spurt, which seems to happen later on [85]. Asthma itself can impair growth through several mechanisms (Table 1). Some of them are directly related to the disease and these include early disease onset, duration and severity of the disease, chest deformity, hypoxemia, impaired pulmonary function, and enhanced metabolic demands due to increased work of breathing and allergic processes. However, up to now, the results on this topic are still conflicting [86–88]. In a population of 121 school-age children with asthma, their weights and heights tended to be similar to those of their peers in those with the first asthma episode before five years of age [89]. Similar results have been confirmed by Murray et al. [90] who performed a study on 183 young Canadians, in which an association between early onset of the disease (before the age of 3 years) and growth retardation has been found. Regarding the severity of asthma, it has been found that children with severe asthma had weight and height below the normal range [89]. In a national study started in 1972 in England and Scotland, an association between the severity of asthma and growth has been observed [91]. In a longitudinal study, McNicol et al. [92] noted a trend towards weight decrease in the group with more severe asthma. However, subsequently, these authors observed growth retardation, mainly for weight, whereas height was affected only in the most extremely severe cases of the disease [93]. Another investigated point is represented by the role of allergic processes. In a study involving children and adolescents with asthma or allergic rhinitis, it has been reported that short stature is more common than expected in atopic children. However, these data were not confirmed by other studies, where no association has been detected between growth defects and the severity of the atopy [94, 95]. Another factor that could be implicated in growth deficiency in children with asthma is represented by the socioeconomic characteristic of youth with asthma. Rona and Florey [91] observed that asthmatic children with lower socioeconomic level showed a higher incidence of short stature. Also Grumach et al. have analyzed the association between socioeconomic level and growth of asthmatic children, confirming that this link was highly significant [96]. Interestingly, it has been largely speculated the role of asthma as a condition inducing growth hormone alterations. In fact, it has been hypothesized that asthmatic children who usually suffer from night time symptoms with sleep disturbance might have an impairment of growth hormone release. Nevertheless, these data have not been confirmed because several other studies demonstrated a normal growth hormone profile in asthmatic children [97].

Up to now, one of the most important factors implicated in the impaired growth of asthmatic children and adolescents is corticosteroids treatment. Due to the inflammatory mechanisms implicated in the pathogenesis of asthma, corticosteroids represent the best way to reduce inflammation of the airways. Inhaled corticosteroids (ICS) represent the cornerstone for the treatment of asthma [98]. At present all guidelines advocate the use of ICS for persistent asthma because they have reduced asthma mortality and morbidity. In addition, they reduce asthma symptoms, improve lung function and reduce the severity of bronchial hyperresponsiveness, and, probably most importantly, reduce the number of exacerbations [99, 100]. However, many side effects were already known, including its effect on growth [101–103]. Of note, also ICS are known to inhibit many key mediators involved in growth: secretion and action of growth hormone, the action of insulin-like growth factors, collagen synthesis, and adrenal androgen production can all be reduced by glucocorticoids [103]. Treatment regimes represent a key factor associated with adrenal suppression in children and adolescents with asthma. In a previous study by Sim et al., high-dose inhaled corticosteroids have been shown to induce adrenal suppression in children with asthma [104]. However, not only high but also low corticosteroids doses (100 mg twice daily) have been shown to have similar effects on adrenal function. In fact, Mohn et al. explored adrenal axis in a small group of 25 children with moderate asthma (16 males/9 females, aged 2–12 years) and treated with inhaled fluticasone propionate with spacer device for up to 3 months. Authors were able to show that low corticoids treatments are associated with adrenal axis suppression during treatment with prompt recovery of the pituitary-adrenal axis once therapy is stopped [105]. However, up to now, the effects of ICS on linear growth and final adult height still remain controversial. In fact, there are several bias related to the effects of ICS on growth during long-term followup: severity of the disease, seasonal variation in growth rate, pubertal status, and socioeconomic factors that might influence growth in subjects with asthma [98].

In prepubertal children, the use of ICS has been shown to reduce growth velocity, resulting in a linear growth reduction of 0.5 to 3.0 cm (approximately 1 cm on average) during the first few years of therapy [106–108]. Although growth velocity returns to normal values within few years after the initiation of ICS therapy, the long-term effect of the initial decrease in growth velocity on attainment of adult height is still unclear [109–111].

In a prospective study, by comparing treatment with budesonide up to 600 *μ*g with sodium cromoglycate, it has been found that ICS did not affect either growth velocity up to 10 years of age or expected final adult height [112]. Interestingly, the incidence of delayed puberty was significantly increased in both groups suggesting that asthma itself, and not ICS per se, has a potential direct influence on growth and onset of puberty. Initial physiological decreased growth velocity could give the impression of growth retardation, but a complete catch-up growth was shown after reaching final adult height. However, one of the main limitations of these studies is that they are not primarily designed to evaluate the effect of ICS on growth. The Childhood Asthma Management program (CAMP) clinical trial was the first study having the main outcome to assess the influence of ICS on growth in a randomized controlled manner [106]. In this study, children were randomly assigned to receive 200 *μ*g budesonide, 8 mg of nedocromil, or placebo twice daily. This study demonstrated in those subjects treated with budesonide a mean increase in height of 1.1 cm less than the mean increase in the placebo group. However, height was still within the normal range based on parental height. In contrast, no difference was documented in terms of height increase between the nedocromil and placebo group. The difference in growth velocity was mainly documented during the first year of treatment and did not increase afterwards.

The available data evaluating the relationship between asthma and growth suffer the influence of the clinical picture, of treatment options, and different study methods that are unable to firmly distinguish those factors which might be responsible for the growth retardation in children and adolescents with asthma. Therefore, further well-designed longitudinal studies are needed to clarify the role of asthma or ICS itself in directly influencing growth and puberty.

## 7. Conclusions

The peculiar onset during childhood of several chronic diseases, mainly T1D, JIA, and asthma requires healthcare planners and providers to consider growth evaluation as one of the main tasks in the regular followup of these populations at high risk of growth impairment. The severity of growth alteration in children and adolescents with chronic diseases is variable, ranging from mild reductions in growth velocity to severe forms leading to an impaired final height. Although specific mechanisms might be involved in each chronic disease, the occurrence of impaired growth may be related to the disease itself or to its related treatment, as well as to a combination of both. These effects result from relevant alteration of growth hormone/insulin like growth factor axis, altering its function at one or more levels, centrally or peripherally or from a direct effect on the growing bone plate (Figure 1).

Allowing regular growth represents one of the main goals in the treatment of children and adolescents with these common chronic diseases. Progress in the understanding of the complex mechanisms related to growth impairment in these chronic diseases has improved the prognosis of growth in these high risk populations, and mainly in children with T1D. Of note, restoring growth alterations by adopting new and specific treatments is now a possible option in these clinical conditions. As early as in the next few years, the development and application of new molecules and therapeutic strategies might prevent and restore all growth abnormalities documented in these high risk populations.

## Figures and Tables

**Table 1:** Main factors associated with the development and progression of bone alterations in children and adolescents with type 1 diabetes, juvenile idiopathic arthritis, and asthma.

Type 1 Diabetes	Juvenile Idiopathic Arthritis	Asthma
(i) Gender (ii) Age at diagnosis (iii) Puberty (iv) Metabolic control (v) Insulin schedules adopted (vi) GH, IGFs, and IGFBPs circulating levels	(i) Degree, extent, and duration of the disease (ii) Age at onset (iii) Corticosteroid treatment (iv) Undernutrition (v) Reduced physical activity (vi) Proinflammatory cytokine levels (interleukin-1*β*, tumor necrosis factor-*α*, and interleukin-6) (vii) Vitamin D metabolites (viii) Sex steroids (ix) Parathyroid hormone-related peptide (x) Fibroblast growth factor (xi) Bone morphogenic proteins (xii) Transforming growth factor superfamily proteins	(i) Age at diagnosis (ii) Duration and severity of the disease (iii) Chest deformity (iv) Hypoxemia (v) Impaired pulmonary function (vi) Enhanced metabolic demands of increased work of breathing (vii) Allergic processes
